# Age-dependent differences in pulmonary host responses in ARDS: a prospective observational cohort study

**DOI:** 10.1186/s13613-019-0529-4

**Published:** 2019-05-14

**Authors:** Laura R. Schouten, Anton H. van Kaam, Franziska Kohse, Floor Veltkamp, Lieuwe D. Bos, Friso M. de Beer, Roosmarijn T. van Hooijdonk, Janneke Horn, Marleen Straat, Esther Witteveen, Gerie J. Glas, Luuk Wieske, Lonneke A. van Vught, Maryse A. Wiewel, Sarah A. Ingelse, Bart Cortjens, Job B. van Woensel, Albert P. Bos, Thomas Walther, Marcus J. Schultz, Roelie M. Wösten-van Asperen, for the MARS consortium, Friso M. de Beer, Lieuwe D. Bos, Gerie J. Glas, Janneke Horn, Arie J. Hoogendijk, Roosmarijn T. van Hooijdonk, Mischa A. Huson, Tom van der Poll, Brendon Scicluna, Laura R. Schouten, Marcus J. Schultz, Marleen Straat, Lonneke A. van Vught, Luuk Wieske, Maryse A. Wiewel, Esther Witteveen, Marc J. Bonten, Olaf L. Cremer, Jos F. Frencken, Kirsten van de Groep, Peter M. Klein Klouwenberg, Maria E. Koster-Brouwer, David S. Ong, Diana M. Verboom

**Affiliations:** 1 Department of Pediatric Intensive Care, Amsterdam University Medical Centers, Amsterdam, The Netherlands; 2 Department of Intensive Care, Amsterdam University Medical Centers, Amsterdam, The Netherlands; 3 Laboratory of Experimental Intensive Care and Anesthesiology (L·E·I·C·A), Amsterdam University Medical Centers, Amsterdam, The Netherlands; 4 Department of Neonatology, Amsterdam University Medical Centers, Amsterdam, The Netherlands; 5 Institute of Medical Biochemistry and Molecular Biology, University Medicine Greifswald, Greifswald, Germany; 6 Department of Pharmacology and Therapeutics, School of Medicine and School of Pharmacy, University College Cork, Cork, Ireland; 7 Center of Experimental Molecular Medicine (CEMM), Amsterdam University Medical Centers, Amsterdam, The Netherlands; 8 Mahidol-Oxford Tropical Medicine Research Unit (MORU), Mahidol University, Bangkok, Thailand; 9 Department of Pediatric Intensive Care, Wilhelmina Children’s Hospital, University Medical Center Utrecht, Lundlaan 6, 3584 EA Utrecht, The Netherlands

**Keywords:** ARDS, Angiotensin converting enzyme, Pathophysiology, Aging, Host response, Biomarkers

## Abstract

**Background:**

Results from preclinical studies suggest that age-dependent differences in host defense and the pulmonary renin–angiotensin system (RAS) are responsible for observed differences in epidemiology of acute respiratory distress syndrome (ARDS) between children and adults. The present study compares biomarkers of host defense and RAS in bronchoalveolar lavage (BAL) fluid from neonates, children, adults, and older adults with ARDS.

**Methods:**

In this prospective observational study, we enrolled mechanical ventilated ARDS patients categorized into four age groups: 20 neonates (< 28 days corrected postnatal age), 29 children (28 days–18 years), 26 adults (18–65 years), and 17 older adults (> 65 years of age). All patients underwent a nondirected BAL within 72 h after intubation. Activities of the two main enzymes of RAS, angiotensin converting enzyme (ACE) and ACE2, and levels of biomarkers of inflammation, endothelial activation, and epithelial damage were determined in BAL fluid.

**Results:**

Levels of myeloperoxidase, interleukin (IL)-6, IL-10, and p-selectin were higher with increasing age, whereas intercellular adhesion molecule-1 was higher in neonates. No differences in activity of ACE and ACE2 were seen between the four age groups.

**Conclusions:**

Age-dependent differences in the levels of biomarkers in lungs of ARDS patients are present. Especially, higher levels of markers involved in the neutrophil response were found with increasing age. In contrast to preclinical studies, age is not associated with changes in the pulmonary RAS.

**Electronic supplementary material:**

The online version of this article (10.1186/s13613-019-0529-4) contains supplementary material, which is available to authorized users.

## Background

Maturation and aging-induced changes in biological pathways involved in the host response to injurious pulmonary and non-pulmonary insults may in part account for observed differences in prevalence and outcome of acute respiratory distress syndrome (ARDS) among different age groups [1]. Preclinical studies using animal models for ARDS have suggested age-dependent differences in pulmonary edema, neutrophil infiltration, and alveolar damage after identical insults [2]. In particular, the pulmonary renin angiotensin system (RAS) seems to play a key role in these differences [3–5]. With increasing age, the balance between the two main enzymes of the pulmonary RAS, angiotensin converting enzyme (ACE) and its natural counteracting enzyme ACE2, shifts toward the lung injurious axis (i.e., ACE), an imbalance that has been associated with aggravating inflammation and increased lung injury [6].

The current challenge is to confirm these findings of animal studies in the human clinical setting. We therefore investigated biomarkers of inflammation, endothelial activation, epithelial damage, and enzymatic activities of the pulmonary RAS in bronchoalveolar lavage (BAL) fluid collected from ARDS patients at different ages. We hypothesized that aging is associated with an intensified host response and a shift in the balance from ACE2 to ACE in ARDS.

## Methods

### Study design

This prospective cohort study was performed at the neonatal, pediatric, and adult intensive care units (ICUs) of the Academic Medical Center, a university hospital in Amsterdam, the Netherlands. This study was conducted as a substudy of the Molecular Diagnosis and Risk Stratification of Sepsis (MARS) project (NCT01905033), a prospective observational cohort study in the adult ICUs of two tertiary teaching hospitals [7, 8]. Patients admitted to the ICU and fulfilling systemic inflammatory response syndrome (SIRS) criteria were included for BAL fluid sampling. To address the current hypothesis, a similar study protocol was used in the neonatal and pediatric ICU patient population (no. NL42386.018.12). Both protocols were approved by the Institutional Review Board, and written informed consent was provided by legal guardians of the patient prior to enrollment. All patients were included between January 2012 and May 2016.

### Inclusion and exclusion criteria

For the present analysis, we included all patients with ARDS, defined according to the criteria stated by the American-European Consensus Conference on ARDS [9]. Based on the updated Berlin Definition for ARDS, we reclassified patients into mild (PaO_2_/FiO_2_ ≤ 300), moderate (PaO_2_/FiO_2_ ≤ 200), and severe ARDS (PaO_2_/FiO_2_ ≤ 100) [10]. To prevent confounding due to differences in ARDS definitions between the age groups, we did not use the recently developed age-specific Pediatric Acute Lung Injury Consensus and Montreux Definition for ARDS [11, 12].

Additional inclusion criteria were two or more of the SIRS criteria on the day of ICU admission and an expected ICU stay of more than 24 h, as these were the two single inclusion criteria of the MARS project. Exclusion criteria were readmission or transfer from another ICU, absence of an arterial line, and receiving antibiotics for more than 48 h prior to admission. Additional exclusion criteria for neonatal and pediatric patients were a body weight of less than 1 kg, immune compromised status, chronic respiratory failure, neuromuscular diseases, cyanotic congenital heart disease, or severe congenital pulmonary abnormalities, and mechanical ventilation within 7 days before eligibility for enrollment into the study. In addition, neonates with postmenstrual age less than 32 weeks and postnatal age less than 7 days were excluded to avoid confounding by neonatal respiratory distress syndrome or transient tachypnea of the newborn.

Patients were stratified into four groups according to age: neonates (< 28 days corrected postnatal age), children (28 days–18 years), adult (18–65 years), and older adults (> 65 years of age).

### Main outcome measures

The primary endpoints were BAL fluid levels of the inflammatory biomarkers interleukin (IL)-6, IL-10, myeloperoxidase (MPO), the endothelial activation markers inter-cellular adhesion molecule (ICAM)-1, vascular endothelial growth factor (VEGF), p-selectin, the markers of epithelial damage clara cell (CC) protein 16, soluble receptor for advanced glycation end-products (sRAGE), and protein levels. In addition, ACE and ACE2 activities in BAL fluid were measured. These markers were chosen based on previous studies indicating that these biomarkers are involved in the main pathophysiological pathways of ARDS and all were reported to be affected by age [2, 13–18].

### Clinical data collection

Demographics, predisposing factors for ARDS, ventilator settings at start of ventilation, oxygenation parameters at onset of ARDS and within the first 24 h after intubation, age-appropriate severity of illness scores [19–21], ICU mortality, and ventilator-free days and alive at day 28 (VFD-28) were recorded. Details on data collection and definitions are described in the Additional file 1.

### BAL fluid sampling and measurements

BAL fluid was obtained within 72 h after intubation. In neonates and pediatric patients, the nondirected lavage was performed with two aliquots of sterile isotonic saline (1 ml/kg; with a maximum of 10 ml) according to guidelines of the European Respiratory Group Taskforce on Bronchoalveolar Lavage in Children [22] and in adults with one aliquot (fixed volume of 15 ml). No estimate of dilution was used in analysis of the data in accordance with the guidelines of the European Respiratory Society (ERS) Task Force Group on Bronchoalveolar Lavage in Children and Adults [22, 23]. A sample was considered invalid if the protein level was below the detection limit of the assay. For details on collection and assays, see the Additional file 1.

### Analysis plan

First, we described baseline characteristic of ARDS patients stratified by the four age groups. Then, we compared the BAL fluid levels of several biomarkers of the host response and ACE and ACE2 activities between the four age groups. To avoid confounding due to differences in dilutions, we also expressed ACE2 and ACE activities as a ratio ACE2/ACE. Finally, we determined the correlations between host response biomarker levels and ACE and ACE2 activities and the ACE2/ACE ratio. A post hoc analysis was performed to explore the effect of severity of ARDS on the relation between the age group and the biomarker levels.

### Statistical analysis

All data are presented as median ± interquartile range (IQR). Group comparisons were evaluated by one-way analysis of variance for normally or Kruskall–Wallis test for non-normally distributed data. A chi-squared test was used for categorical variables. Group differences for host response biomarker levels and ACE and ACE2 activities were tested by post hoc Dunn’s test with Bonferroni correction for multiple comparisons. The correlations between ACE, ACE2 activity, ACE2/ACE ratio, and biomarkers were tested by the Spearman’s rho test. The effect of ARDS severity on the association between age and the biomarker levels was explored by linear regression models, with the age group as the independent variable, the biomarkers as the dependent variable and PaO_2_-to-FiO_2_ ratio as a potential confounder.

Statistical analysis was performed using R-statistics 2.15.0 (R Foundation, Vienna, Austria, www.r-project.org). A *p* value less than 0.05 was considered statistically significant.

## Results

### Clinical characteristics of patients

**Fig. 1:**
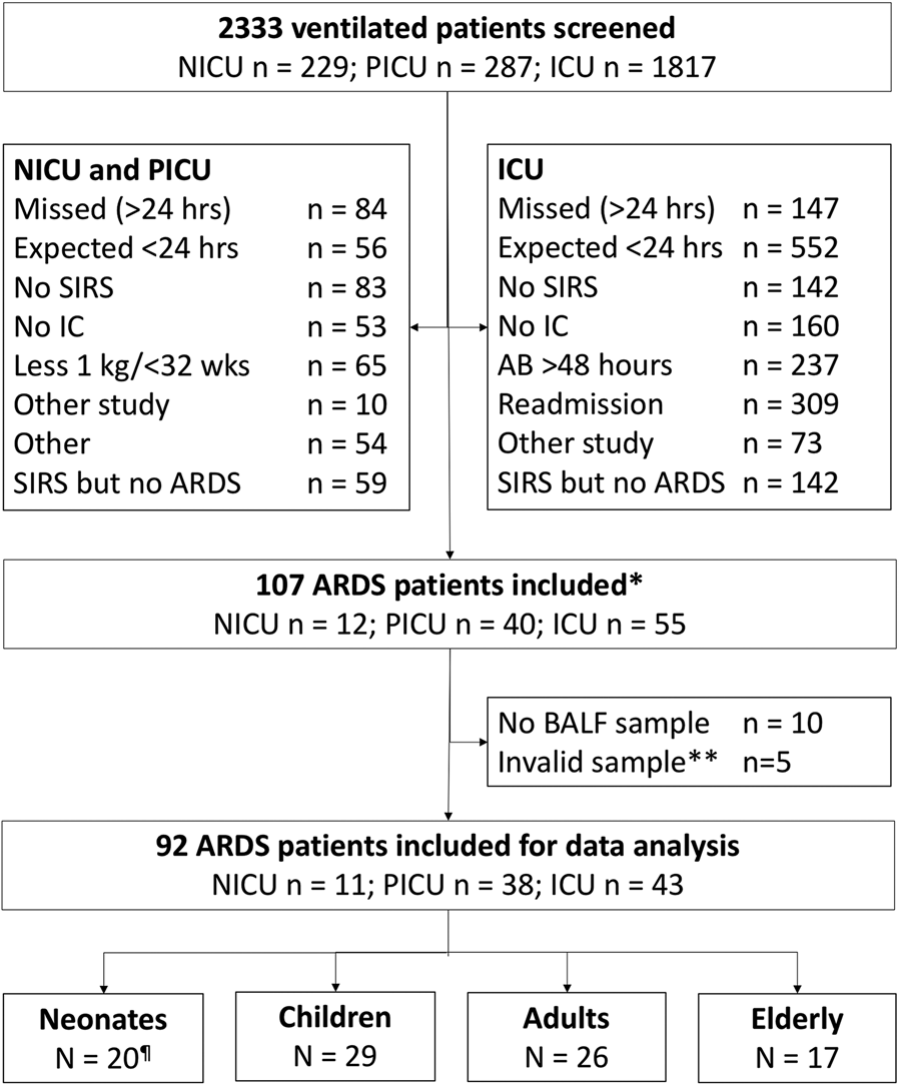
Flowchart. * ARDS was defined by the Berlin definition; ** A sample was considered invalid if no proteins could be measured. ARDS = acute respiratory distress syndrome ^¶^ 1 premature neonate, 36 weeks at time of inclusion. The exclusion criteria were not exclusive. In case a patient fulfilled more than one exclusion criteria, only one was chosen to report. *AB* antibiotics, *ARDS* acute respiratory distress syndrome, *BALF* bronchoalveolar lavage fluid, *No IC* no informed consent, *SIRS* systemic inflammatory response syndrome

We enrolled 92 ARDS patients (Fig. 1). Patient characteristics are presented in Table 1. In all four age groups, ARDS was considered to have a direct cause in most cases. A detailed description of predisposing factors is provided in Additional file 1: Table S1.

**Table 1:** Baseline characteristics and outcome

Variables	Neonates	Children	Adults	Older adults	*p* value
	*N* = 20	*N* = 29	*N* = 26	*N* = 17	
Demographics					
Age (years), median [IQR]	0.03 [0.00–0.05]	0.21 [0.12–0.82]	55 [48–61]	74 [70–77]	NA
Male, *n* (%)	10 (50)	20 (62)	14(54)	9 (53)	0.85
Race, (Caucasian), *n* (%)	18 (90)	25 (86)	20 (77)	15 (88)	0.64
Severity of illness score, median [IQR]	16 [5–22]^a^	4.4 [4.0–5.0]^b^	85 [66–125]^c^	87 [79–106]^c^	NA
Predisposing factors^d^					
Direct hit, *n* (%)	14 (70)	27 (93)	16 (61)	12 (70)	< 0.001
Indirect hit, *n* (%)	6 (30)	2 (7)	10 (39)	5 (30)	0.09
Oxygenation					
PaO_2_/FiO_2_ at onset, median [IQR]	88 [64–129]	137 [115–190]	124 [83–153]	146 [85–182]	0.03
PaO_2_/FiO_2_ after 24 h, median [IQR]	187 [138–229]	188 [140–227]	188 [150–215]	205 [177–281]	0.41
Berlin classification					
PaO_2_/FiO_2_ 200-300 (mild), *n* (%)	2 (12)	3 (9)	3 (12)	4 (24)	0.58
PaO_2_/FiO_2_ 100-200 (moderate), *n* (%)	4 (24)	26 (81)	14 (54)	8 (47)	0.002
PaO_2_/FiO_2_ < 100 (severe), *n* (%)	11 (65)	3 (9)	9 (35)	5 (29)	0.001
Study procedure					
Timing BAL from time of ARDS diagnosis, days, median [IQR]	1 [0–1]	1 [0–1]	1 [0–1]	1 [0–1]	0.56
At start of ventilation					
Tidal volume (ml/kg), median [IQR]	7.0 [5.0–7.3]	6.9 [5.6–7.9]	6.6 [4.9–7.8]	5.1 [4.5–6.1]	0.07
PEEP (cmH_2_O), median [IQR]	7 [6–8]	6 [5–7]	8 [5–10]	10 [8–12]	0.002
High-frequency oscillation, *n* (%)	10 (50)	0 (0)	0 (0)	0 (0)	NA
Outcome					
Mortality at ICU, *n* (%)	2 (10)	0(0)	11 (42)	3 (18)	< 0.001
VFD and alive at day 28*, days, median [IQR]	21 [18–25]	18 [17–20]	17 [0–24]	21 [15–25]	0.18

Most neonates (65%) were classified as having severe ARDS, while the proportion of severe ARDS was much lower among the other age groups (9%, 35%, and 29% in children, adults, and older adults, respectively). The median PaO_2_-to-FiO_2_ ratio at onset of ARDS was significantly lower in neonates when compared to children, adults, and older adults. Of note, after 24 h, median PaO_2_-to-FiO_2_ ratio increased in all age groups and there were no differences between the age groups anymore.

In general, tidal volumes (V_T_), when expressed in ml/kg ideal body weight, were comparable among the four age groups. Independent of age, patients were all ventilated with V_T_ < 8 ml/kg ideal body weight. In contrast, PEEP was significantly higher in adults and older adults when compared to neonates and children. In neonates, high-frequency oscillation (HFO) ventilation was most frequently used.

Mortality rates differed significantly between the age groups (*p* < 0.001), with the highest mortality rate in adults (48%) and no mortality in children (Table 1).

### Host response biomarker levels in BAL fluid

**Fig. 2:**
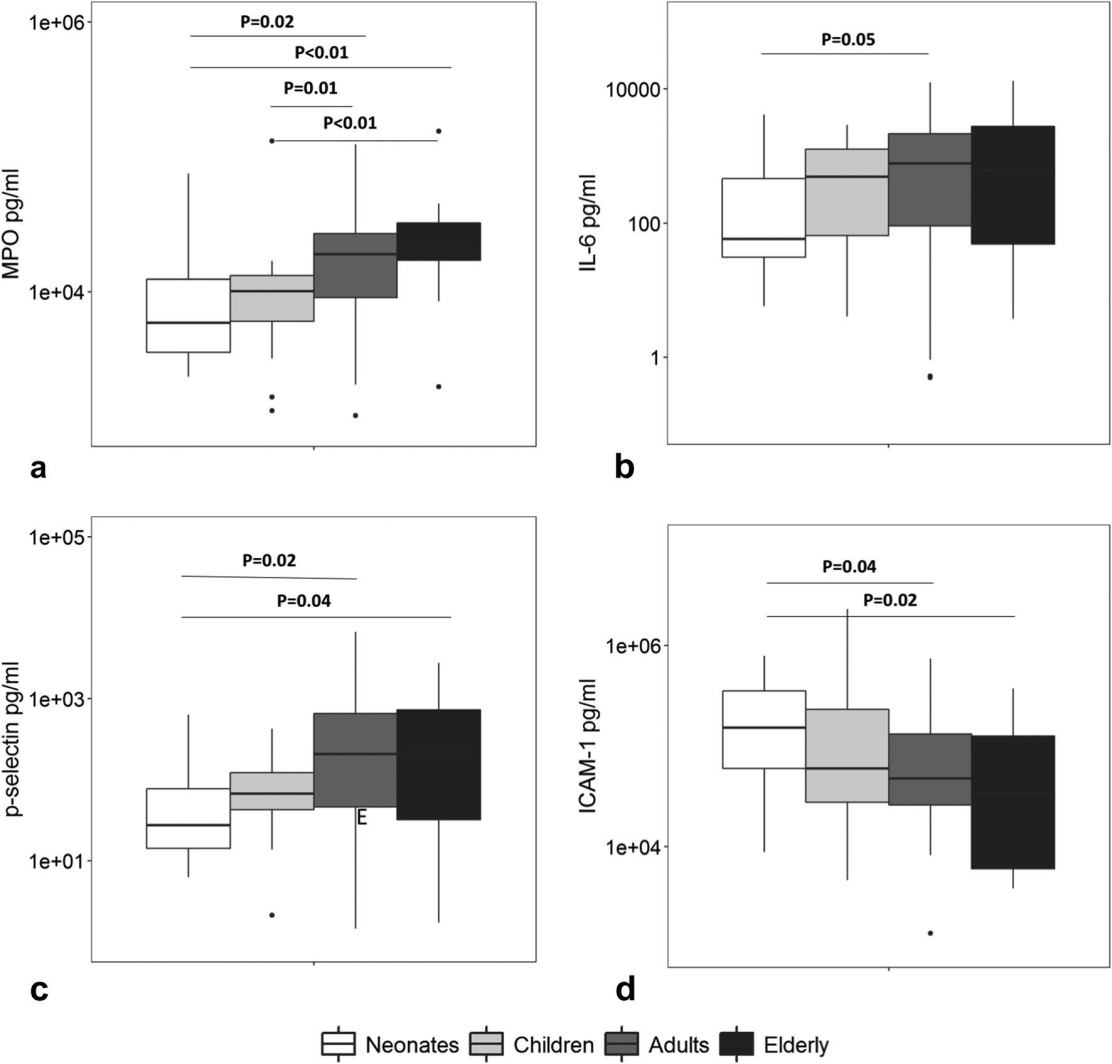
Markers of inflammation, endothelial activation and epithelial activation in bronchoalveolar lavage fluid of ARDS patients. **a** Myeloperoxidase (MPO), **b** interleukin (IL)-6, **c** p-selectin, **d** intercellular adhesion molecule (ICAM)-1 levels in bronchoalveolar lavage (BAL) fluid of ARDS patients stratified by four age groups. Horizontal bars represent the median. Group differences were tested with a Dunn’s test with Bonferroni correction for multiple comparisons. A *p* value less than 0.05 was considered statistical significant

BAL fluid levels of MPO were significantly lower in neonates and children when compared to adults and older adults (Fig. 2a). In addition, significant lower levels of IL-6, IL-10, and p-selectin were found in neonates when compared to the other age groups (Fig. 2b, c, Additional file 1: Fig. S1a). Protein levels and CC-16 levels showed a similar trend, albeit not significant after correction for multiple testing (Additional file 1: Fig. S1c and d). Interestingly, the endothelial activation marker ICAM-1 showed an opposite trend, with significantly higher levels in neonates compared to adults and older adults (Fig. 2d). VEGF and sRAGE showed no age-related differences (Additional file 1: Fig. S1b and e). A post hoc analysis showed that the association between increasing age and increased levels of MPO, IL-10, p-selectin and the decreased levels of ICAM-1 remained significant after correction for severity of ARDS (Additional file 1: Table S3).

### BAL fluid ACE and ACE2 activities

**Fig. 3:**
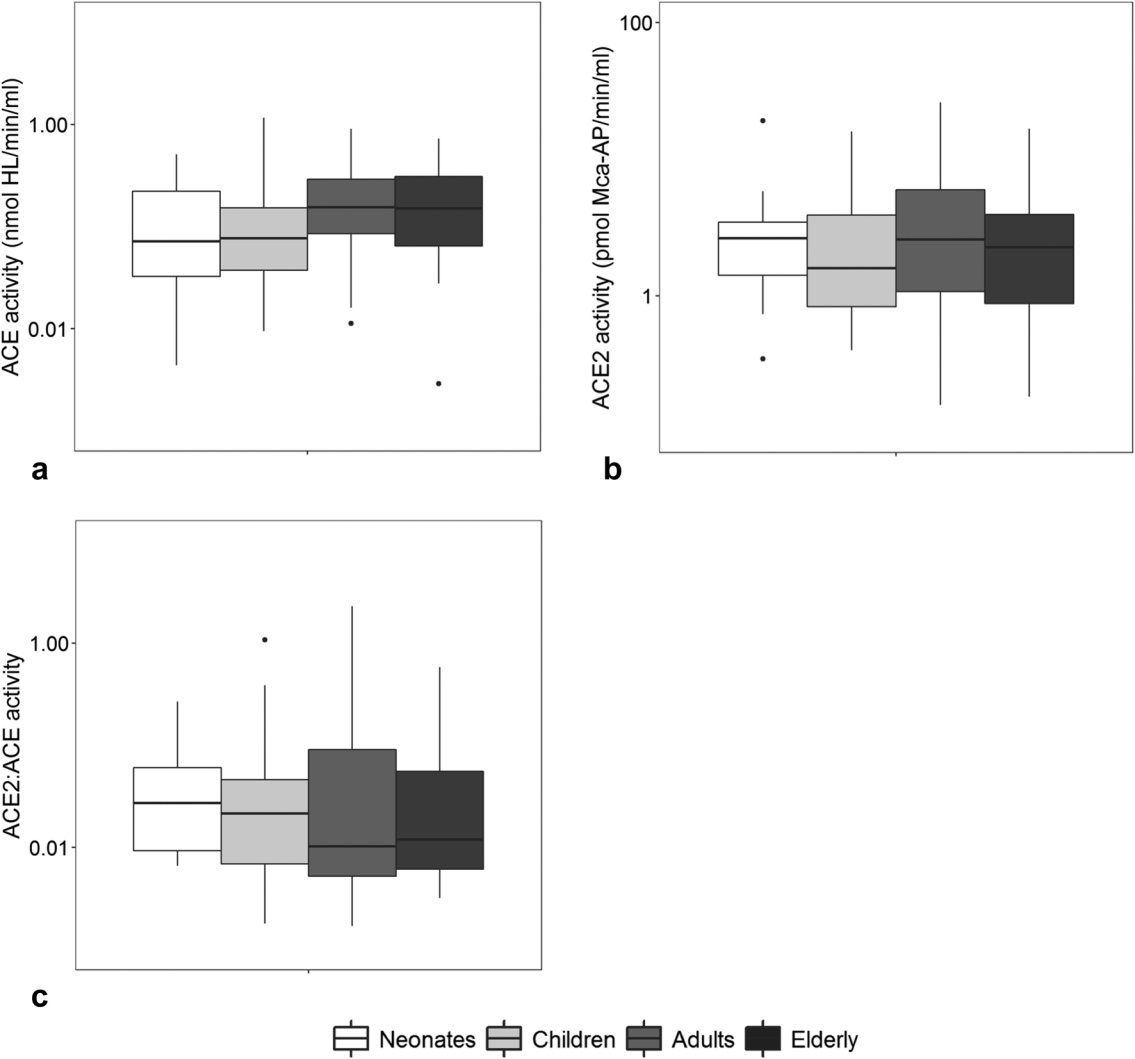
ACE and ACE2 activity in bronchoalveolar lavage fluid of ARDS patients. **a** ACE and **b** ACE2 activity in bronchoalveolar lavage (BAL) fluid of ARDS patients stratified by four age groups. **c** ACE2/ACE ratio. Horizontal bars represent the median. Group differences were tested with a Dunn’s test with Bonferroni correction for multiple comparisons. A *p* value less than 0.05 was considered statistical significant. *ACE* angiotensin converting enzyme

Comparison of BAL fluid ACE activities (Fig. 3a), ACE2 activities (Fig. 3b), and ACE2/ACE ratios (Fig. 3c) between the four age groups revealed no significant differences. Independent of age, the ACE2/ACE ratio showed a weak correlation with protein levels (rho = 0.30, *p* < 0.01). There were no correlations between the ACE2/ACE ratio and the host response biomarkers (Additional file 1: Table S2). There was a weak positive correlation between ACE activities and increased levels of the inflammatory markers MPO, IL-6, epithelial damage marker sRAGE and endothelial activation marker p-selectin, while ACE2 showed a weak positive correlation with increased levels of the endothelial activation markers ICAM-1, VEGF, and p-selectin (Additional file 1: Table S2).

## Discussion

In this study, we found an increase in pulmonary inflammatory mediators involved in the neutrophil response with increasing age. No age-dependent differences in ACE and ACE2 were seen, suggesting that the age-dependent differences in the pulmonary host response are not mediated by changes in pulmonary RAS.

This is the first study in which the pulmonary host response in ARDS is compared between patients of different ages. It is a unique cohort as we included not only children and adults, but also neonates and older adults, clinically important subgroups of ARDS patients who are frequently excluded from clinical studies. Mechanical ventilation practice was applied according to protocols stressing lung-protective ventilator settings, and we strictly followed standardized operating procedures to obtain BAL fluid.

We found age-dependent differences, especially in markers known to be involved the neutrophil response (MPO, IL-6, and IL-10), one of the hallmarks of ARDS [24]. Levels of these markers were significantly lower in neonates/children compared to adults/older adults. Preclinical studies also showed that neutrophil influx into the lung and the accompanying inflammatory mediator response is lower in neonatal and juvenile animals compared to adult and older animals [2, 15, 25–27]. A murine model of ARDS showed differences in the activation of genetic networks of several inflammatory pathways (e.g., cell migration and interleukin activity) involved in the pathophysiology of ARDS between juvenile and adult animals [15]. Synergistic interactions between pulmonary injurious hits, i.e., LPS and mechanical ventilation, were observed in the adult animals as a result of coordinated changes in gene expression of these pathways. These interactions did not occur in the juveniles. Maturational differences in the innate immune system are predominately studied in neonates, while fewer data exist from older children. In neonates, the absolute number of neutrophils is significantly lower than in adults due to immaturity of the proliferative pool and they demonstrate reduced responsiveness and diminished extravasation [28]. It is suggested that the latter is caused by a lower expression of adhesion molecules, such as p-selectin and less production of chemotactic mediators by resident inflammatory cells [28]. Indeed, we found lower levels of the adhesion molecule p-selectin and the chemotactic cytokines (IL-6, IL-10) in the BAL fluid of neonates with ARDS. Moreover, these levels tended to increase with increasing age. In septic neonates, children and adults with similar age-dependent differences in levels of adhesion molecules have been described in blood plasma [29].

In contrast, we found the highest levels of the other adhesion molecule ICAM-1 in neonates. ICAM-1 is an adhesion molecule on endothelial cells and is known to play an important role in neutrophil tracking into the lung [28]. Levels of adhesion molecules in BAL fluid are the result of both protein expression and proteolytic activity of sheddases [29]. P-selectin and ICAM-1 are known to be cleaved by different enzymes, and age-related differences in proteolytic activity of sheddases have been reported [30, 31]. This may account for the discrepancy we found between p-selectin and ICAM-1 levels. Moreover, in our study, neonates had the lowest PaO_2_-to-FiO_2_ ratio, which is known to increase levels of ICAM-1 [32].

In contrast to the evidence in preclinical studies, our data could not confirm an age-dependent role of the pulmonary RAS in the development and course of ARDS [6]. An important reason may be the wide individual variation in the levels of ACE and ACE2 activities within the age groups. This could have interfered with the possibility to detect small, but clinically relevant, age-related differences. A recent clinical trial showed similar variability of RAS activation in adult ARDS patients [33] and speculated that this may be caused by both intrinsic (i.e., genetic) variability of RAS activity and heterogeneity of ARDS [34]. In addition, our study included only those patients who had already developed ARDS and needed to be ventilated, while in preclinical studies, enzyme activities are measured just a few hours after the initial insult. Temporal changes in RAS activation are known to exist [35]. Above all, independent of the age groups, we found no correlation between ACE2/ACE ratio and the host response biomarkers. Moreover, ACE and ACE2 activities were only weakly correlated with some of the inflammatory markers and endothelial activation markers. This suggests that the pulmonary RAS may not be the leading pathway in the inflammatory response in humans with ARDS. It must be noted that preclinical studies investigating ARDS have repeatedly failed in their translation into clinical practice and these models may not reflect the complex pathophysiology of ARDS in patients [36]. Accordingly, studies have shown that the host responses in murine models correlate poorly with human conditions [37]. As a consequence, usefulness and applicability of these models are an important matter of debate [38].

Our study has several limitations. Although the largest study ever, the sample size per age group was still relatively small. This could explain, at least in part, the lack of differences between the three age groups. Indeed, several age-dependent differences in biomarker levels became nonsignificant after correction for multiple testing. This suggests that the current study may have been underpowered. Second, differences in dilution of the lavage fluid may have influenced the results. Studies have indicated that an important part of the variability in fluid recovery is due to the way lavage fluid is obtained [22, 23]. Therefore, only trained physicians could perform the lavage in this study, and a standardized operating procedure was strict followed. According to the guidelines of the European Respiratory Group Taskforce on Bronchoalveolar Lavage in Children, we did not correct for the effects of dilution or return yield in our analyses [22]. There is currently no reliable indicator which can be used for correction [22]. Therefore, we expressed the pulmonary RAS enzymatic activities as a ratio to avoid confounding. However, BAL fluid levels of the host response biomarkers could still have been influenced by dilution differences. Though, based on the opposite directions of the effects of age found in levels of the different biomarkers, these age-related differences found are unlikely to be fully explained by differences in dilution. Third, we investigated only biomarker levels in the alveolar compartment. Therefore, we were not able to determine whether the differences in biomarker concentrations are due to age-related differences in the local host response or maybe caused by leakage and reflect age-related variation in the systemic host response. Finally, we applied the Berlin definition of ARDS in all age groups and not the Pediatric Acute Lung Injury Consensus criteria for pediatric [11] or the Montreux criteria for neonatal ARDS [12] to prevent bias due to differences in the definition of ARDS. There are important discrepancies between the Berlin definition and these age-specific criteria which might have given another distribution of the ARDS severity classes. But, this would not have influenced our outcomes, i.e., levels of host response biomarkers and RAS enzyme activities.

## Conclusions

In conclusion, this study found differences in BAL fluid levels of biomarkers involved in the neutrophil response between the age groups, which may form an explanation for the previously observed age-related differences in epidemiology of ARDS. However, no differences in the pulmonary RAS were found between the different age groups. This suggests a role for other underlying pathophysiological mechanisms. Understanding the age-related differences in the host response during ARDS is essential for the development of effective therapy.

## Additional file

**Additional file 1.** Methods - Detailed description of the two study protocols Methods - Collected data and definitions Methods - Sample collection and assays. **Table S1**. Predisposing factors. **Figure S1**. Markers of inflammation, endothelial activation and epithelial activation in bronchoalveolar lavage fluid of ARDS patients. **Table S2**. Correlation between ACE, ACE2 activity, ACE2:ACE ratio and biomarkers of inflammation, endothelial activation and epithelial damage. **Table S3**. Association between age-groups and biomarkers in bronchoalveolar lavage fluid of ARDS patients.

## Data Availability

The datasets used and/or analyzed during the current study are available from the corresponding author on reasonable request.

## Abbreviations

ACE: angiotensin converting enzyme
ARDS: acute respiratory distress syndrome
BAL: bronchoalveolar lavage
CC: clara cell
ICAM: intercellular adhesion molecule
HFO: high-frequency oscillation
ICU: intensive care unit
IL: interleukin
IQR: interquartile range
MARS: Molecular Diagnosis and Risk Stratification of Sepsis
MPO: myeloperoxidase
PEEP: positive end expiratory pressure
RAS: renin–angiotensin system
SIRS: systemic inflammatory response syndrome
sRAGE: soluble receptor for advanced glycation end-products
VEGF: vascular endothelial growth factor
VFD-28: ventilator-free days and alive at day 28
V_T_: tidal volume
