# Supplementary material for: Age-dependent differences in pulmonary host responses in ARDS: a prospective observational cohort study
Source: Ann Intensive Care. 2019 May 14;9:55. doi: 10.1186/s13613-019-0529-4 (PMC6517452; doi:10.1186/s13613-019-0529-4)
Supplement: Supplementary file 1 — Additional file 1. Methods - Detailed description of the two study protocols Methods - Collected data and definitions Methods - Sample collection and assays. Table S1. Predisposing factors. Figure S1. Markers of inflammation, endothelial activation and epithelial activation in bronchoalveolar lavage fluid of ARDS patients. Table S2. Correlation between ACE, ACE2 activity, ACE2:ACE ratio and biomarkers of inflammation, endothelial activation and epithelial damage. Table S3. Association between age-groups and biomarkers in bronchoalveolar lavage fluid of ARDS patients. [file 13613_2019_529_MOESM1_ESM.docx]

**Additional file**

**Age–dependent Differences in Pulmonary Host Responses in ARDS – a prospective observational cohort study**

Laura R. Schouten^1,2,3^; Anton H. van Kaam^4^, Franziska Kohse ^5,6^, Floor Veltkamp^1^, Lieuwe D. Bos^2,3^, Friso M. de Beer^2,3^, Roosmarijn T. van Hooijdonk^2,3^, Janneke Horn^2,3^, Marleen Straat, MD^2,3^, Esther Witteveen^2,3^, Gerie Glas^2,3^, Luuk Wieske^2,3^, Lonneke A. van Vught^7^, Maryse A. Wiewel^7^, Sarah A. Ingelse^1^, Bart Cortjens^1^, Job B. van Woensel^1^, Albert P. Bos^1^, Thomas Walther^5,6^, Marcus J. Schultz^2,3,8^, Roelie M. Wösten-van-Asperen^1,9^, for the MARS consortium*

^1^Department of Pediatric Intensive Care, Amsterdam University Medical Centers, the Netherlands

^2^Department of Intensive Care, Amsterdam University Medical Centers, the Netherlands

^3^Laboratory of Experimental Intensive Care and Anesthesiology (L·E·I·C·A), Amsterdam University Medical Centers, the Netherlands

^4^Department of Neonatology, Amsterdam University Medical Centers, the Netherlands

^5^Institute of Medical Biochemistry and Molecular Biology, University Medicine Greifswald, Germany

^6^Department of Pharmacology and Therapeutics, School of Medicine and School of Pharmacy, University College Cork, Cork, Ireland

^7^Center of Experimental Molecular Medicine (CEMM), Amsterdam University Medical Centers, the Netherlands

^8^Mahidol–Oxford Tropical Medicine Research Unit (MORU), Mahidol University, Bangkok, Thailand

^9^Department of Pediatric Intensive Care, Wilhelmina Children’s Hospital, University Medical Center Utrecht, the Netherlands

**MARS consortium members:**

Academic Medical Center, Amsterdam, The Netherlands: Friso M. de Beer, Lieuwe D. Bos, Gerie J. Glas, Janneke Horn, Arie J. Hoogendijk, Roosmarijn T. van Hooijdonk, Mischa A. Huson, Tom van der Poll, Brendon Scicluna, Laura R. Schouten, Marcus J. Schultz, Marleen Straat, Lonneke A. van Vught, Luuk Wieske, Maryse A. Wiewel, and Esther Witteveen

University Medical Center Utrecht, Utrecht, The Netherlands: Marc J. Bonten, Olaf L. Cremer, Jos F. Frencken, Kirsten van de Groep, Peter M. Klein Klouwenberg, Maria E. Koster–Brouwer, David S. Ong, and Diana M. Verboom

ONLINE MATERIAL

**Methods** - Detailed description of the two study protocols

**Methods** - Collected data and definitions

**Methods** - Sample collection and assays

**Table 1** - Predisposing factors

**Figure 1** - Markers of inflammation, endothelial activation and epithelial activation in bronchoalveolar lavage fluid of ARDS patients

**Table 2** - Correlation between ACE, ACE2 activity, ACE2:ACE ratio and biomarkers of inflammation, endothelial activation and epithelial damage

**Table 3** – Association between age-groups and biomarkers in bronchoalveolar lavage fluid of ARDS patients.

**METHODS**

Detailed description of the two study protocols from which the present cohort is selected:

**Protocol no NL34294.018.10**:

Setting: Adult intensive care unit (ICU), Academic Medical Center of Amsterdam.

Inclusion criteria:

- Enrolment within 24 hours after admission.
- Expected to stay more than 24 hours
- Two or more of the systemic inflammatory response syndrome (SIRS) criteria on the day of ICU admission
  - Body temperature <36°C or >38°C
  - Tachycardia >90/min
  - Tachypnea >20/min or PO2 <4. kPa
  - Leucocyte count <4 x 109/L or >12 x 109/L
- Signed informed consent

Exclusion criteria:

- Readmissions or transfer from other ICUs
- Receiving antibiotics for more than 48 hours prior to admission
- The absence of an arterial line
- Inclusion in another study

**Protocol no. NL42386.018.12:**

Setting: Neonatal intensive care unit (NICU) or pediatric intensive care unit (PICU) of the Emma Children’s Hospital, Academic Medical Center of Amsterdam.

Inclusion criteria:

- Intubated and mechanically ventilated, with an anticipated duration of mechanical ventilation of at least 24 hours at enrolment
- Two or more of the following criteria at the time of enrolment.
  - Hypothermia (< 36 °C), fever (> 38.0 °C), or body temperature instability.
  - Bradycardia or tachycardia, according to age, or a prolonged capillary refill time.
  - Tachypnea or apnea’s, according to age, or cyanosis.
  - Leucopenia or leucocytosis, according to age, or more than 10 % banded neutrophils.
- Signed informed consent

Exclusion criteria:

- Neonates with a postmenstrual age less than 32 weeks and a postnatal age less than 7 days.
- Immune compromised; chronic respiratory failure, neuromuscular diseases, cyanotic congenital heart disease or signs of cardiogenic pulmonary edema, severe congenital pulmonary abnormalities.
- Receiving antibiotics for more than 48 hours prior to admission

**Collected data and definitions**

Age-appropriate severity scores were collected. Neonatal Acute Physiology Score for neonates [1], Pediatric Index of Mortality 2 for children [2], and Acute Physiology and Chronic Health Evaluation IV score for adults and older adults [3]. Predisposing factors for ARDS were dichotomized to either ‘direct’ (pneumonia, aspiration, meconium aspiration) or ‘indirect’ (sepsis, trauma, pancreatitis, asphyxia, necrotic enterocolitis) (4-6). The number of VFD–28 was defined as the number of days between successful weaning from invasive mechanical ventilation and day 28 after study enrolment. Patients who died before day 28 received zero VFD–28. Non–invasive mechanical support was not considered as mechanical ventilation.

**Sample collection and assays**

BAL fluid was immediately centrifuged (1500g, 15 min, 4°C) and stored at -80ºC. The panel of inflammatory biomarkers, endothelial activation markers and markers of epithelial damage were analyzed simultaneously using a Luminex kit according to the manufacturer instructions (Luminex; R&D systems, Minneapolis, USA). BAL fluid ACE and ACE2 activities were fluorimetrically measured as previously described [7, 8]. BAL fluid protein levels were quantified by the Pierce BCA-Protein Assay Kit Thermo Scientific, USA.

Reference:

1. Richardson DK, Corcoran JD, Escobar GJ, et al. SNAP-II and SNAPPE-II: Simplified newborn illness severity and mortality risk scores. J Pediatr. 2001; 138:92–100.

2. Slater A, Shann F, Pearson G, et al. PIM2: a revised version of the Paediatric Index of Mortality. Intensive Care Med. 2003;29:278–85.

3. Zimmerman JE, Kramer AA, McNair DS, et al. Acute Physiology and Chronic Health Evaluation (APACHE) IV: hospital mortality assessment for today’s critically ill patients. Crit Care Med. 2006;34:1297–310.

4. Calfee CS, Janz DR, Bernard GR, et al. Distinct molecular phenotypes of direct vs indirect ARDS in single-center and multicenter studies. Chest. 2015;147:1539–1548.

5. De Luca D, van Kaam AH, Tingay DG, et al. The Montreux definition of neonatal ARDS: biological and clinical background behind the description of a new entity. Lancet Respir Med. 2017;5:657–666.

6 Ranieri VM, Rubenfeld GD, Thompson BT, et al. Acute respiratory distress syndrome: the Berlin Definition. JAMA. 2012;307:2526–33.

Pediatric T, Lung A, Consensus I, et al. Pediatric acute respiratory distress syndrome: consensus recommendations from the Pediatric Acute Lung Injury Consensus Conference. Pediatr Crit Care Med. 2015;16:428–39.

7. Schwager SL, Carmona AK, Sturrock ED. A high-throughput fluorimetric assay for angiotensin I-converting enzyme. Nat Protoc. 2006;1:1961–4.

8. Vickers C, Hales P, Kaushik V, et al. Hydrolysis of biological peptides by human angiotensin-converting enzyme-related carboxypeptidase. J Biol Chem. 2002;277:14838-43.

**Table 1. Predisposing factors**

|  | **Neonates** | **Children** | **Adults** | **Older adults** |
| --- | --- | --- | --- | --- |
| **Direct** | 14 (70) | 27 (93) | 16 (61) | 12 (70) |
| Aspiration, n (%) | 0 (0) | 0 (0) | 2 (7) | 0 (0) |
| MAS, n (%) | 6 (30) | NA | NA | NA |
| Pneumonia, n (%) | 8 (40) | 27 (93) | 14 (54) | 12 (70) |
| **Indirect** | 6 (30) | 2 (7) | 10 (39) | 5 (30) |
| Asphyxia, n (%) | 1 (6) | NA | NA | NA |
| Trauma, n (%) | 0 (0) | 1 (3) | 0 (0) | 1 (6) |
| Sepsis, n (%) | 5 (25) | 1 (3) | 10 (39) | 3 (18) |
| Pancreatitis, n (%) | 0 (0) | 0 (0) | 0 (0) | 1 (6) |

MAS, meconium aspiration syndrome; NA, not applicable

**Figure 1. Markers of inflammation, endothelial activation and epithelial activation in bronchoalveolar lavage fluid of ARDS patients** **
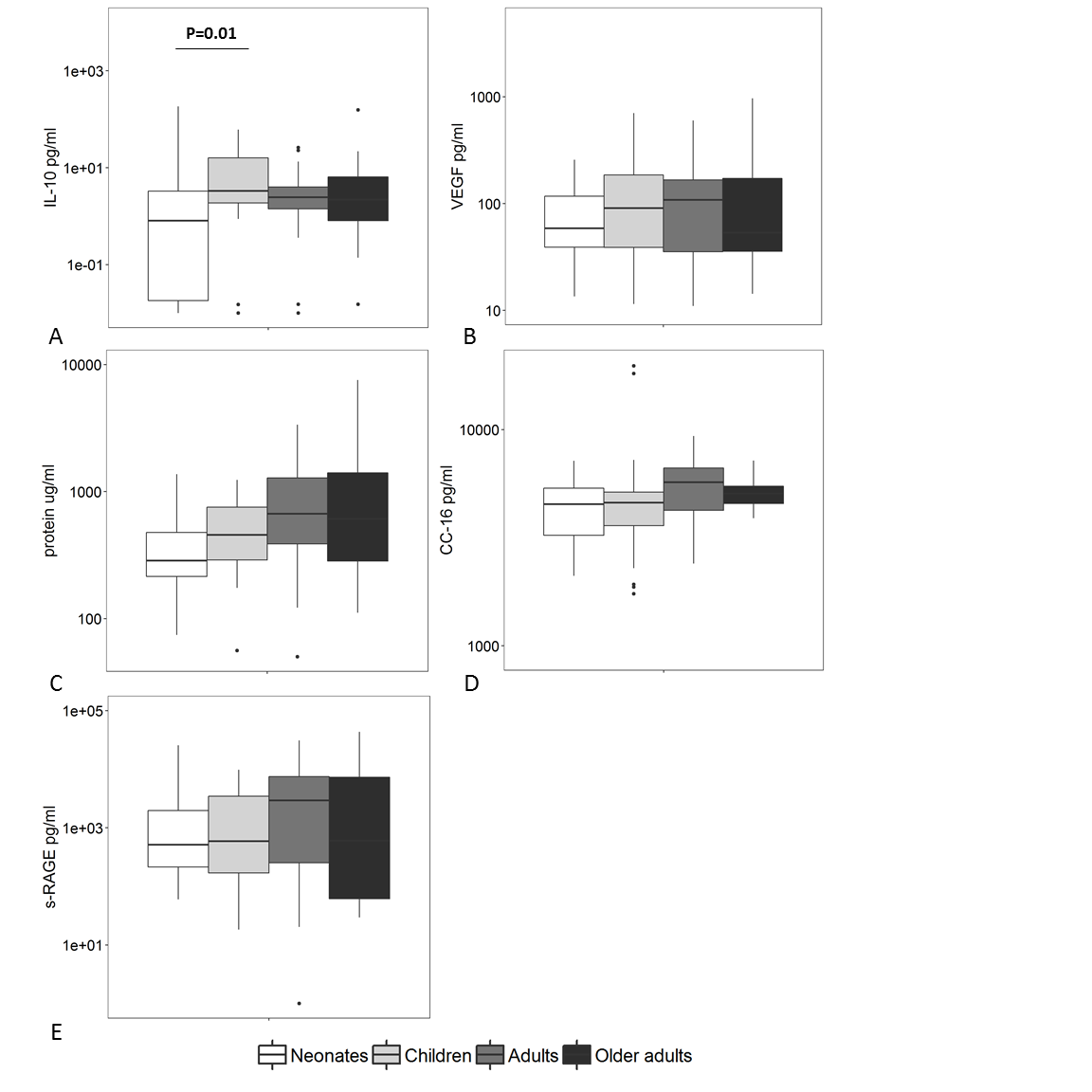
**

(A) interleukin–10, (B) vascular endothelial growth factor (VEGF) (C) protein (D) clara cells (CC)–16, (E) soluble receptor for advanced glycation end products (sRAGE) levels in bronchoalveolar lavage (BAL) fluid of ARDS patients stratified by four age groups. Horizontal bars represent the median. Group differences were tested with a Dunn test with Bonferroni correction for multiple comparisons. A p-value less than 0.05 was considered statistical significant.

**Table 2. Correlation between ACE, ACE2 activity, ACE2:ACE ratio and biomarkers of inflammation, endothelial activation and epithelial damage.**

|  | **ACE activity** | **ACE2 activity** | **ACE2:ACE** |
| --- | --- | --- | --- |
| **PaO_2_:FiO_2_** | -0.10 | -0.14 | 0.05 |
| **Inflammatory marker** |  |  |  |
| **MPO** | 0.27* | 0.08 | -0.05 |
| **IL-6** | 0.27* | 0.34* | 0.10 |
| **IL-10** | 0.21* | 0.08 | -0.12 |
| **Endothelial activation** |  |  |  |
| **ICAM-1** | 0.23* | 0.32* | 0.18 |
| **p-selectin** | 0.35* | 0.38* | 0.09 |
| **VEGF** | 0.20* | 0.32* | 0.10 |
| **Epithelial damage** |  |  |  |
| **Protein** | 0.23* | 0.58* | 0.30* |
| **CC-16** | 0.24* | 0.14 | 0.00 |
| **sRAGE** | 0.39* | 0.20* | 0.01 |

Rho values from 0 to 0.25 or from 0 to -0.25 indicate the absence of correlation (white), r values from 0.25 to 0.50 or from -0.25 to -0.50 are considered weak a correlation (light grey), r values ranging from 0.50 to 0.75 or -0.50 to -0.75 (dark grey) indicate a moderate to good correlation, and r values from 0.75 to 1 or from -0.75 to -1 point to very good to excellent correlations (1. Dawson B, Tra­pp RG. Basic and Cli­nical Biostatistics. 4th Ed. New York: Lange Medical Books/McGraw-Hill; 2004.)

* p value less than 0.05 was considered statistical significant. ACE = Angiotensin converting enzyme; CC = clara cell; ICAM = intercellular adhesion molecule; IL = interleukin; VEGF = vascular endothelial growth factor; MPO = myeloperoxidase; sRAGE = soluble receptor for advanced glycation end-products.

**Table 3. Association between age-groups and biomarkers in bronchoalveolar lavage fluid of ARDS patients.**

|  | **Unadjusted** | | **Adjusted for PaO_2_:FiO_2_** | |
| --- | --- | --- | --- | --- |
| **MPO** | **ß-coefficient** | **p-value** | **ß-coefficient** | **p-value** |
| Neonates | Reference |  | Reference |  |
| Children | -0.09 | 0.82 | 0.16 | 0.69 |
| Adults | 0.92 | 0.02 | 1.01 | <0.01 |
| Older adults | 1.36 | <0.01 | 1.51 | <0.01 |
| **IL-6** | **ß-coefficient** | **p-value** | **ß-coefficient** | **p-value** |
| Neonates | Reference |  | Reference |  |
| Children | 0.98 | 0.13 | 1.16 | 0.11 |
| Adults | 0.98 | 0.14 | 1.05 | 0.11 |
| Older adults | 1.07 | 0.14 | 1.18 | 0.28 |
| **IL-10** | **ß-coefficient** | **p-value** | **ß-coefficient** | **p-value** |
| Neonates | Reference |  | Reference |  |
| Children | 1.94 | <0.01 | 1.97 | <0.01 |
| Adults | 1.32 | 0.04 | 1.33 | 0.04 |
| Older adults | 1.33 | 0.06 | 1.35 | 0.06 |
| **ICAM-1** | **ß-coefficient** | **p-value** | **ß-coefficient** | **p-value** |
| Neonates | Reference |  | Reference |  |
| Children | -0.60 | 0.21 | -0.57 | 0.25 |
| Adults | -1.30 | <0.01 | -1.29 | 0.01 |
| Older adults | -1.38 | 0.02 | -1.36 | 0.01 |
| **p-selectin** | **ß-coefficient** | **p-value** | **ß-coefficient** | **p-value** |
| Neonates | Reference |  | Reference |  |
| Children | 0.53 | 0.32 | 0.62 | 0.27 |
| Adults | 1.10 | 0.05 | 1.14 | 0.04 |
| Older adults | 1.29 | 0.04 | 1.35 | 0.03 |
| **VEGF** | **ß-coefficient** | **p-value** | **ß-coefficient** | **p-value** |
| Neonates | Reference |  | Reference |  |
| Children | 0.36 | 0.35 | 0.27 | 0.83 |
| Adults | -0.05 | 0.90 | -0.08 | 0.58 |
| Older adults | 0.30 | 0.49 | 0.24 | 0.38 |
| **Protein** | **ß-coefficient** | **p-value** | **ß-coefficient** | **p-value** |
| Neonates | Reference |  | Reference |  |
| Children | 0.32 | 0.39 | 0.46 | 0.23 |
| Adults | 0.30 | 0.43 | 0.35 | 0.36 |
| Older adults | 0.55 | 0.20 | 0.42 | 0.14 |
| **CC-16** | **ß-coefficient** | **p-value** | **ß-coefficient** | **p-value** |
| Neonates | Reference |  | Reference |  |
| Children | 0.06 | 0.80 | 0.56 | 0.82 |
| Adults | -0.13 | 0.58 | -0.14 | 0.59 |
| Older adults | 0.21 | 0.43 | 0.21 | 0.45 |
| **s-RAGE** | **ß-coefficient** | **p-value** | **ß-coefficient** | **p-value** |
| Neonates | Reference |  | Reference |  |
| Children | 0.12 | 0.86 | 0.07 | 0.39 |
| Adults | 0.51 | 0.45 | 0.58 | 0.79 |
| Older adults | 0.08 | 0.91 | 0.20 | 0.27 |
| **ACE activity** | **ß-coefficient** | **p-value** | **ß-coefficient** | **p-value** |
| Neonates | Reference |  | Reference |  |
| Children | 0.14 | 0.94 | 0.00 | 0.99 |
| Adults | 0.07 | 0.27 | 0.06 | 0.30 |
| Older adults | 0.08 | 0.25 | 0.08 | 0.27 |
| **ACE2 activity** | **ß-coefficient** | **p-value** | **ß-coefficient** | **p-value** |
| Neonates | Reference |  | Reference |  |
| Children | -0.24 | 0.88 | -0.39 | 0.81 |
| Adults | 2.17 | 0.18 | 2.11 | 0.19 |
| Older adults | 0.72 | 0.68 | 0.63 | 0.73 |
| **ACE2:ACE** | **ß-coefficient** | **p-value** | **ß-coefficient** | **p-value** |
| Neonates | Reference |  | Reference |  |
| Children | 0.04 | 0.67 | 0.05 | 0.55 |
| Adults | 0.13 | 0.13 | 0.14 | 0.12 |
| Older adults | 0.03 | 0.77 | 0.04 | 0.70 |

Separate linear regression models to assess the association between the age groups and the biomarkers. The levels of biomarkers of inflammation, epithelial damage and endothelial activation were log transformed to obtain a normal distribution. Neonates were used as the reference group. ACE = Angiotensin converting enzyme; CC = clara cell; ICAM = intercellular adhesion molecule; IL = interleukin; VEGF = vascular endothelial growth factor; MPO = myeloperoxidase; sRAGE = soluble receptor for advanced glycation end-products. A p-value less than 0.05 was considered statistical significant.
